# First Nations and Metis peoples’ access and equity challenges with early childhood oral health: a qualitative study

**DOI:** 10.1186/s12939-021-01476-5

**Published:** 2021-06-07

**Authors:** Grace Kyoon-Achan, Robert J. Schroth, Daniella DeMaré, Melina Sturym, Jeannette M. Edwards, Julianne Sanguins, Rhonda Campbell, Frances Chartrand, Mary Bertone, Michael E. K. Moffatt

**Affiliations:** 1 Department of Preventive Dental Science, Dr. Gerald Niznick College Of Dentistry, Rady Faculty of Health Sciences, University of Manitoba, Winnipeg, Manitoba R3E 3P4 Canada; 2 Children’s Hospital Research Institute of Manitoba, Winnipeg, Canada; 3 Ongomiizwin Research – Indigenous Institute of Health and Healing, Rady Faculty of Health Sciences, University of Manitoba, Winnipeg, Manitoba R3E 3P4 Canada; 4 Department of Community Health Sciences, Max Rady College of Medicine, Rady Faculty of Health Sciences, University of Manitoba, Winnipeg, Canada; 5 Department of Pediatrics and Child Health, Max Rady College of Medicine, Rady Faculty of Health Sciences, University of Manitoba, Winnipeg, Canada; 6 Shared Health Manitoba, 1610-155 Carlton St, Winnipeg, MB R3C 3H8 Canada; 7 First Nations Health and Social Secretariat of Manitoba, 600-275 Portage Ave, Winnipeg, MB R3B 2B3 Canada; 8 Health & Wellness Department, Manitoba Metis Federation, 150 Henry Avenue, Winnipeg, MB R3B 0J7 Canada

**Keywords:** Early childhood caries, Early childhood Oral health, Indigenous, First Nations, Metis, Access, Equity

## Abstract

**Background:**

Inequities in early childhood oral health are evident amongst Indigenous peoples and communities in Manitoba, Canada. Early childhood caries (ECC) is decay in primary dentition in children under 6 years of age. A severe form of the disease occurs at a higher rate in Indigenous populations compared to the general population. ECC has been strongly associated with social determinants of health.

**Methods:**

Focus groups and sharing circles were conducted with four First Nations and Metis communities in urban and rural communities in Manitoba. There were eight groups in total of purposively sampled participants (*n* = 59). A grounded theory approach guided thematic analysis of audio recorded and transcribed data.

**Results:**

Indigenous participants experienced challenges similar to those found in the general population, such as encouraging and motivating parents and caregivers to establish regular oral hygiene routines for their children. However other challenges reported, disproportionately affect Indigenous communities. These include poor access to dental care, specifically no dental offices within 1 h driving radius and not having transportation to get there. Not having evidence-based oral health information to support good oral hygiene practices, preventing parents from making the best choices of oral hygiene products and oral health behaviours for their children. Poverty and food insecurity resulting in poor nutritional choices and leading to ECC. For example, feeding children sugary foods and beverages because those are more readily avialble than healthy options. Confusing or difficult encounters with dental professionals, highlighted as a factor that can erode trust, reduce compliance and impact continued attendance at dental offices.

**Conclusion:**

Closing existing early childhood oral health gaps for First Nations and Metis peoples and communities requires equity-oriented healthcare approaches to address specific problems and challenges faced by these populations. Family, community and systemic level interventions that directly implement community recommendations are needed.

## Background

Early childhood caries (ECC) continues to be highly prevalent globally [1, 2]. ECC is caries involving the primary teeth in children < 6 years of age. The high prevalence of ECC, among Indigenous people globally reflects social injustice, socio-economic inequalities and systemic inequities [3]. In Canada, First Nations, Inuit and Metis children experience higher rates of ECC [4, 5] than the rest of the population, with nearly 1 in every 4 children affected by a severe form of the disease, known as severe early childhood caries (S-ECC) [6]. Indigenous children also account for a high proportion of children receiving rehabilitative dental surgery under general anesthesia (GA) [6, 7]. It has been suggested that the single biggest risk factor for caries in this population is poverty affecting diet, especially frequent sugar consumption combined with poor oral hygiene habits [8, 9]. Populations that are more prone to ECC, which include Indigenous peoples, face challenges that include parents’ own poor oral health status, parenting practices such as not firmly implementing tooth brushing, lack of dental insurance, which may create a lack of access to dental services, the experience of racism, and social determinants of health [10–12].

First Nations (FN) and Metis children in Manitoba Canada experience high rates of ECC due to limited factors such as income, education, rural and remote dwelling reducing access to care in urban centers, and lack of amenities [13]. We conducted focus groups and sharing circles to fully understand community-based experiences, inequities and challenges articulated by individuals and families living within their local realities in order to provide contextual and tailored oral health interventions and promotion [14]. The goal is to promote equity oriented healthcare that is designed to address specific local contexts and realities. This has been shown to increase patient confidence in healthcare and improve health outcomes [15].

Dental care is currently not part of Canada’s universal health care program. For the majority of Canadians, dental care is privately-funded and delivered on a fee-for-service basis [16, 17]. Unlike other provinces, Manitoba lacks a provincial children’s dental program targeting low-income families. Most Canadians get 80–100% coverage through employment insurance or receive basic or limited dental care through government programs, including employment and income assistance and the non-insured health benefits (NIHB) program. Status Indians (i.e. First Nations people) registered under the Indian Act [18] and recognized Inuit persons receive dental benefits from the NIHB program [19, 20].

Dental coverage through NIHB for eligible FN and Inuit peoples include diagnostic, preventive, restorative, endodontic, periodontal and prosthodontic services, oral surgery, limited orthodontic and adjunctive dental services, with the exception of extensive rehabilitation and cosmetic treatments. Metis peoples, however, are not covered under the NIHB, leaving this population without appropriate access to oral healthcare when they do not have employment-based private insurance and are not on social assistance. Even with the limited NIHB coverage for dental services, FN and Metis peoples alike face difficulties in accessing dental care, albeit differently. While Metis peoples simply do not have federally or provincially covered dental care, FN with NIHB coverage are often unaware of or confused about what services are covered under the NIHB program. Overall, we consider that the NIHB program has improved over the years and has removed a lot of the predetermination processes that were previously required. However, a real issue is that some beneficiaries and dental offices may be unaware of enhancements and improvements, as most offices are not automatically notified of the changes being made [21]. Moreover, services providers may also be oblivious to the challenges posed by limited social determinants of health and system-imposed barriers to accessing oral health care faced by Indigenous peoples in Manitoba.

The purpose of this article is to report the challenges and problems faced by FN and Metis parents in meeting the early childhood oral health (ECOH) needs of their children and to offer context-based and community informed recommendations on improving oral healthcare equity and outcomes in FN and Metis communities in Manitoba.

## Methods

This was a community-based participatory research (CBPR) study. The CBPR approach has been previously successfully applied to engage Indigenous peoples and communities [22]. CBPR is a relationships based, decolonizing methodology that emphasizes respectful and mutually beneficial partnerships with Indigenous peoples and communities [23, 24]. The study built on the already existing foundation of an oral health promotion initiative in Manitoba. The Healthy Smile Happy Child (HSHC) initiative is a multidisciplinary and intersectoral partnership working to prevent ECC among children in Manitoba, including Indigenous children [25]. Its activities are guided by community engagement and development, knowledge exchange, and evaluation to ensure ongoing quality improvement. Among other strategies, the initiative works to promote community awareness and acceptance of ECOH, partnerships with existing community-based programs to deliver ECOH promotion and sustained ECC prevention activities in community-based settings.

Partnership was with the key organizations that represent First Nations and Metis peoples in Manitoba, the First Nations Health and Social Secretariat of Manitoba (FNHSSM), the Manitoba Metis Federation (MMF) and the communities that participated in the study.

Building relationships from the outset, intentionally positions communities for ownership and implementation of actionable strategies for better ECOH [26]. Community partners helped design study questions, identified study sites, coordinated the setting up of focus groups in culturally appropriate and respectful ways, explained and took the lead on local engagement protocols and reviewed data, manuscripts and knowledge translation materials created from the results. Ethics approvals for the study were obtained from the University of Manitoba’s Health Research Ethics Board. Other approvals were received from the Health Information Research Governance Committee at the FNHSSM and also from the MMF.

A grounded theory (GT) approach was used to facilitate understanding of participants’ experiences with ECOH in their communities. This process meant that data collection and analysis were concurrently conducted and emerging themes used in subsequent data collection sessions [27]. There was constant comparison, a key element in GT approach, where interviews were conducted, preliminarily analyzed, compared with previous findings and then applied in guiding the next round of data collection. Concepts and themes were constructed from the experiences and perspectives shared by participants in the study [28].

Focus groups and sharing circles were used to collect data. Sharing circles are a traditional way to respectfully conduct conversations in Indigenous cultures. They are rooted in oral traditions, are storied and explore individual and collective perspectives on a subject matter [29]. Standard focus groups with larger emphasis on facilitation, were used in Metis communities [30]. Interviews were semi-structured and encouraged in-depth discussion of and group thinking on the subject matter. Participants were free to reframe the questions to better address relevant issues pertaining to their local contexts and realities, or to respond by adding new insights that the questions may not have specifically assumed. There were a total of eight groups: four were in an urban setting and four were in rural communities. Two of the groups primarily comprised FN participants, two were with Metis participants, and four mixed groups comprised both FN and Metis participants.

Participants were purposively sampled and recruited by community-based oral health promoters and program leads. Participants in all groups were Indigenous community members, parents, and grandparents of children less than 72 months old. Focus groups and sharing circles were facilitated by the university-based research team and led by an experienced qualitative researcher. All participants provided written consent forms, members of the research team went over the forms in details and responded to any questions from any participants who had questions or requested more clarification while completing the forms. Meals were provided during the sessions that lasted between three and 6 h. Participants also received gift cards, tooth brushes, toothpastes and flossers in appreciation for their time and participation. This is never to be construed as payment for information, but as respect for participant’s contribution. Fuller details of the method and process have been described [30, 31].

There were 10 key and one general discussion questions used in the interview guide [See Table 1]. However, only two questions relating to challenges faced by parents are considered for this article: 1) what are some of the problems and challenges you face when taking care of your child’s teeth? and 2) are there any stories you would like to share about your child or children’s dental experiences?

**Table 1:** Interview Questions

Study questions	
1	Do you think healthy baby teeth make a difference in the child’s overall health?
2	Do you think what you eat and how you take care of your teeth while pregnant will affect your child’s teeth?
3	What do you think makes young kids get cavities or decay in their baby teeth?
4	How and why do you to take care of your children’s teeth? a. What are some of the things you currently do to take care of your baby’s or young child’s teeth? b. What are (other) things you could do?
5	Where did you learn about baby teeth and how to take care of them? a. What or who influenced what you now know about taking care of baby’s teeth? b. Did you find these resources respectful of your cultural traditions?
6	What do you think are the best ways to get important information and tips to parents and families to keep kids’ teeth cavity-free and avoid getting serious tooth decay? Are there any particular places where you would like to see more information about children’s dental health? Where and from whom?
7	**What are some of the challenges or problems you face when taking care of your child’s teeth?** **Has this led to any problems regarding their dental health (e.g. tooth decay)?**
8	What are your thoughts about getting dental work done under general anesthesia? a. Do kids in your community go to have dental surgery in the operating room? How did it make you feel? b. Why do you think so many kids go to the operating room*?*
9	Some people say tooth decay can be prevented, even in young children. What do you think about this?
10	What is the best way to get oral health information across to you?
11	**Any stories you would like to share with us about your child or children’s dental experiences?**

Participant responses were audio recorded and transcribed verbatim. Data were analyzed by the lead qualitative researcher and two program staff who had also assisted with the focus groups and sharing circles. Data was analyzed together using an open coding system for an overall sense of the challenges faced by all participants, and then FN and Metis data were considered separately to note any differences between both data sets. Preliminary results were shared with all community partners for feedback and input. Further thematic analysis was completed using Nvivo 12 software™. Quotes indicate FN and Metis identity to indicate distinction of thought.

## Results

A total of 59 FN and Metis community members, ranging in age from 21 to 71 years participated in the study. There were a few male participants (18.4%/*n* = 11) and 81.6% (*n* = 48) of all participants were females. While about 44% of all participants said they were employed, only 20% reported having employer sponsored dental insurance. Some FN participants said they had NIHB coverage and 35% reported not having any insurance coverage. The majority (88%) of participants said they had children, others were grandparents with grandchildren under 6 years of age. About half of all participants were (50%) were married or living in common-law relationships. Actual numbers in all categories are shown in [Table 2].

**Table 2:** Participants’ Characteristics

Variable	N (%)
Indigenous Group	
First Nations	32 (54.2%)
Metis	27 (45.8%)
Employment Status	
Employed/Maternity Leave	26 (44.7%)
Unemployed	33 (55.93%)
Marital Status	
Married	13 (22.03%)
Single	25 (42.39%)
Common-Law	16 (35.59%)
No Answer	5 (8.47%)
Educational Level	
Completed University	1 (1.69%)
Some post-secondary	23 (38.98%)
Some high school	16 (27.12%)
Completed High School	14 (23.73)
Other/NA	5 (8.47%)
Parenting Status	
Not having children	7 (11.86%)
Have children < 6 years old	52 (88.14%)
Insurance status	
Employer sponsored insurance	12 (20.34%)
Non-insured health benefits	15 (25.42%)
Employment and income assistance	11 (18.64%)
No insurance or not sure	21 (35.59%)

Overall 10 themes emerged and include: difficulties scheduling dental appointments, the high cost of dental care and insurance difficulties, lack of oral health information and a lack of available oral hygiene products. Food insecurity and poor nutrition, transportation difficulties, unhelpful parental or family members’ behaviours, children’s behaviours, fear, and a distrust of dentists were also highlighted.

### Difficulty scheduling dental appointments

Participants reported difficulties when attempting to access dental care for their young children, especially when parents have several children. This reportedly posed scheduling and travel related difficulties, particularly for rural dwelling families.

I think it’s the big families. The parents are aware that their kids have tooth decay or they are having tooth problems. They need to see a dentist, but it’s really hard to get into a dentist when you have six kids. There is no way you are going to get six kids; they are going to put you one-week, next week, the next one the next week, and like who wants to be at the dentist that many times … But being in this area where there are no dentists here, you have to travel out of town to see the dentist, so that’s another issue - FN participant (rural community)

Some disappointments like you're booking appointments, these appointments take forever to get, like you can call for an appointment … I don't know if any of you guys tried to call children's dental offices to make the damn appointment … they're literally months down the road. If you missed that appointment, you're screwed. Your kid is not seeing the dentist for over a year because of the wait times. It doesn't matter what location you're trying to get to. You can say, look at all the appointment times that are open at every location and give me the soonest one. You're still waiting. Oh, it's such a hassle – Mixed group participant (urban community)

Families can become discouraged and abandon efforts to provide their children dental care when they have multiple children needing dental care, with appointments being as far off as 6 months away. Parental discouragement and consequent inaction can expose children at risk children to early onset of tooth decay, and possibly worsening tooth decay.

### Cost of dental care and insurance difficulties

Participants expressed difficulties paying for dental care and having no or inadequate dental coverage.

Well, being First Nation you automatically have proof of (NIHB) payment but only for a cleaning every six months. But then if you have insurance then [dentists] want you there every time, but if you don’t have coverage they don’t care how your kids are … So if I didn’t have insurance, well I need lots of money to take my kids to the dentist. My little sister, same boat, she has no money, no insurance so she can’t take her kids to the dentist. - FN participant (rural community)

What I find is there's a lot of assumptions made by healthcare providers that everybody's covered under First Nation’s health. But what I would like to say is people are working in minimum wage jobs, a lot of those jobs don't come with benefits, so they don't have benefits through work. They're making just enough money or not on income assistance so you don't have any benefits through there. So they have no coverage and even things like [dental care] that should not be an issue, it becomes an issue. – Metis participant (rural community)

Yes, I actually know somebody who they have issues with their teeth because they don’t have any insurance, they are not on social assistance, they are not status, and they are not on nothing so it is very difficult for them. They haven’t really been to the dentist at all - Mixed group participant (urban community)

Participants who are not able to afford dental care and who do not have dental insurance coverage were less likely to seek out dental care. Participants also said they knew several others experiencing similar problems in the communities.

### Lack of information

Participants said that inadequate information hampers parental action that could improve ECOH.

I don’t want to say that we live in poverty, but our people don’t have access to the internet, they don’t have TV where they can see these things [dental information], all they have relied on is the health department [in the community] for where they can find this information and lots of people don’t access it. Even in our community right now, there are a couple of parents that have many, many children, but they don’t receive anything. – FN participant (rural community)

With my son, I didn’t know how to take care of his teeth, he was two years old I had to be at work and for a drink, I’d gave him juice. I had no idea that apple juice is not supposed to be good for you and that’s what I gave him. The milk that he had was [canned] milk – Metis participant (rural community)

Part of it, I think, would have to do with the fact that a lot of people don't know when to take your child to the dentist for the first time. So like my daughter just turned one, and I thought that it was at three [years], and I just happen to be in the dentist’s [office] and there's all those signs and they said at one [year of age] – Mixed group participant in urban community

A lack of information is reported to be the reason for parental lack of action to prevent ECC. Some parents were unaware of the effects of sugary drinks on teeth or the seriousness of untreated tooth decay. Families reported not having avenues (Internet or TV) to access information on caring for their children’s oral health, which contributes to seeming inaction on their part. Others did not know at what age to begin taking their children for dental assessment and a few assumed they could manage dental cavities by brushing them away and risked unabated progression of cavities.

### Lack of oral hygiene products

Participants said that not being able to afford oral hygiene products makes it challenging for parents to care for their children’s teeth.

Well maybe provide the parents with the little brushes … once they [children] start getting teeth. When you’re First Nations you’re budgeted. Then who is going to drive all the way to [town] and go buy a little toothbrush and toothpaste, you know … That’s the only solution I would see … – FN participant (rural community)

Few problems I have is seeing my son run out of toothpaste. There is no store here [in the community] so you can’t just go walking down the street and go pick up a toothpaste or a toothbrush or floss. You have to wait until you can go to town or get somebody that's going to town and give them money to bring it. It's hard not to have access to toothbrush and toothpaste and fluoride just like that. I pretty much boil his toothbrush and I try and keep it as long as I can. That's one of my challenges – Metis participant (rural community)

We don't have a store to buy stuff. Sometimes you could use maybe baking soda or salty water. Maybe water to brush your teeth – Metis participant (rural community)

I think it depends too on how and what's in your budget if you can afford [toothpaste and tooth brushes] sometimes. You go over your budget and sometimes you forgo to buy toothpaste or toothbrush – Mixed group participant (urban community)

Both rural and urban dwelling parents reported difficulties with a lack of oral hygiene products. While the rural dwelling parents pointed to not having a store to replenish products, urban dwelling parents talked about relegating oral hygiene products in favour of food and other life sustaining needs.

### Food insecurity and poor nutrition

Poverty and inability to afford healthier food options was also reported. Parents resort to cheaper less healthy foods when they are not able to afford better options.

People don’t have enough money to buy healthy food, milk and vegetables, they’re limited with money... So [parents] are buying junk food, because they can’t afford the good stuff. We don’t have the hunters we used to have to bring the ducks, the wild meat that doesn’t rot our teeth. Like fish, that’s what we need to be eating – FN participant (rural community)

Both me and my husband work and if he goes to see a dentist it's going to cost us $400 and that $400 would put groceries in our fridge, we choose groceries or dentist and right now that's not like an option – Metis participant (rural community)

One of the problems that families face in the community would definitely be poverty. When you’re lower income you don’t have all the stuff to be able to get all these things done. So that affects taking the [children] to the dentist or buying healthier food for them to eat – Mixed group participant (urban community)

There are just all sorts of things … there's other important bills that come before [dental care] you know the roof over your head and like some people are struggling – Mixed group participant (urban community)

Parents indicated that they are faced with choosing overall survival versus oral health, with the latter suffering. Many said that poverty forces them to choose basic needs over oral hygiene products. Therefore, early childhood oral health is compromised by cost of living and a lack of quality nutrition.

### Transportation difficulties

Participants revealed that transportation is a significant barrier to accessing dental care as is also making alternative care arrangements to travel outside the community for dental care.

You have to get a cab. Every week you have to get a cab. You have to get a babysitter to watch the other five kids because you are only able to take in one at a time. So I think that’s a problem here too – FN participant (rural community)

And transportation is an issue. Like we have three kids now and we have another one on the way … not everybody's going to have the ability to get a vehicle that’s big enough to transport them. [A colleague] she's got six kids plus herself. So for her to find a vehicle that were even capable of carrying seven children is a big expense. It’s just a huge cost and transportation is a major barrier in this community for dental care. If you are not First Nations you don’t qualify for [Medical Transportation] unless you are on income assistance – Metis participant (rural community)

I think maybe something that is also a factor, is transportation because a lot of people don't have vehicles. So in order to make a dentist appointment, it's about taking the five minutes out of your day to book the appointment. Then it takes the time to remember these appointments and then it takes the time to think of the process of getting to these appointments. So some people might not be able to handle stress … - Mixed group participant (urban community)

Transportation is clearly an ongoing barrier to accessing dental care for both urban and rural dwelling participants. Difficulties in this area for either group is due to poverty or a lack of financial wherewithal.

### Parental behaviours

Some parents reported difficulties in taking care of their children’s teeth, such as not having the time or when they have children with special healthcare needs.

Some people just don’t even know how to make appointments for themselves. They never have to do that and then they have children and they don’t even know how to do that for their children because they never did it for themselves. Some people have like six or eight kids and they don’t even worry about that. They are too busy just getting by every day, making food to eat. - FN participant (rural community)

The other issue that I have is my oldest son is six and has Spina Bifida so I also have issues with his hand motor control not being what it should be for his age. So that also becomes an issue. If you have a child with any kind of special needs, that just adds another layer of things – Metis participant (rural community)

This [brushing children’s teeth] is a parenting skill! It's just what I lack. I think if you [parent] have a lack in taking care of your teeth so then it's kind of hard trying to be there for your child as they grow up … And then it doesn't become something so important to them because you don't press them too much - Mixed group participant (urban community)

Parents reported difficulties developing and consistently executing oral hygiene routines for their children. This appears to be especially hard when children are living with disabilities or when parents work evening or late shifts and are not physically present to enforce or supervise teeth brushing.

### Behaviours of other family members

Participants also recounted challenges with curtailing potentially harmful behaviors of other family members or friends concerning their children’s oral care.

Well in my family my parents like to spoil my son, so they like to give him goodies. They like to give him apple juice in a bottle and I try to step in normally [but] they try to sneak it in. So I try to educate everybody and even people who aren’t parents - FN participant (rural community)

Some kids, they don’t have someone to teach them how to do it [brush] right or help them do it. I tell my boys, but I have family members that their kids have never been to a dentist or they don’t brush their teeth and their parents don’t help to try to make them. They don’t like brushing kids’ teeth. There needs to be more education for parents to get to realize how important it is - FN participant (rural community)

Grandparents are great, but sometimes are a problem, I'll tell my kids “no, you can't have candy” and Mama and Papa will show up and bring something … they bring cookies and we say we don’t give them those but they just give them – Metis participant (rural community)

Participants said they face difficulties caring for children’s teeth when other family members do not share their knowledge or share the same values and attitudes about ECOH. As children spend time with other family members, education for the entire family and even neighbours may be necessary so that more adults around the child are aware of good oral health habits.

### Children’s behaviours

Participants pointed out difficulties with establishing oral care for uncooperative children, saying that some children dislike brushing and make oral care a challenging task for parents.

Well the child also just forgets or doesn’t want to brush their teeth. They run away, they hide, they cry. They just refuse to brush their teeth. But we really try our best to take care of her teeth. There are nights where she doesn't want to brush but I sit her on the potty and then like, we’ll brush her teeth and then they go to bed. It's how we do this. And with couples, its communication right? Ask did you brush their teeth? Make sure you brush their teeth. I always tell my fiancé because I am not the only one putting [the child] to bed. - Mixed group participant (urban community)

I was thinking that another reason that some kids don’t like to brush is because when I was little I had a severe sensitive gag reflex and sometimes it’s really bad. You can actually hit their gag reflex doing that [brushing]. So sometimes I would think that for younger kids that is probably what it is, is the gag reflex. I also think my girls are stubborn. My girls are very stubborn and very head strong, so that’s a big challenge for me. For me, it’s my girls’ stubbornness is what keeps us back more than anything else - Mixed group participant (urban community)

When my kids were smaller they hated their teeth being brushed and being a single mom is really hard because you’ve got to hold them down, you have to figure a way to hold them down and brush their teeth for them. It was very difficult. After a while I learned to wrap them in a blanket so they can’t move their arms and then that’s how I brushed their teeth - Mixed group participant (urban community)

Dealing with uncooperative children was said to leave parents exhausted and make oral care an unexciting chore. Participants mentioned that couples working together may reduce the burden. Parents used creative ways to ensure they are properly caring for their children’s teeth or seek to understand what biological reasons may be making brushing hard for the child such a strong gag reflex.

### Fear and distrust of dentists

Several participants indicated that they questioned dentists’ motives as they had less than straightforward encounters with dental providers, resulting in fear and distrust in all such cases.

I took my kids to the dentist because I had coverage from insurance, so they would take my kids, tell them they need this, they need spacers, they need whatever, but as soon as I didn’t have insurance, they didn’t even call me again. So I think it has a lot to do with money too. They are pulling your kids teeth out for nothing sometimes, so I told my kids to take better care of their teeth, brush their teeth morning and bedtime [to avoid dentists] - FN participant (rural community)

They don’t go to the dentist because some people are scared of dentists. I know some parents are. They don’t want to have to deal with it, you know. They are just running away from it - FN participant (rural community)

I hate to say it but personal experience, I kind of felt that the dentist rushed for it right away because like I said, when I was at a private place, the first thing, he [dentist] just kind of took a look in his [son’s] mouth and then right away he was like, “oh he needs surgery”. Not even like 15 seconds he was in his mouth and right away he was like, he needs surgery. And then my daughter who's three who loves the dentist, she's also opening up [her mouth], you know, and he's like, oh, “she's going to need surgery” … I was like hell no, I knew, I'm not even going to deal with this … Then [a different] dentist told me straight up that he [my son] doesn't need surgery. Those teeth were being pushed out. She [dentist] is like those are all going to fall out. Those are just his baby teeth. But the other dentist was like, oh, he needs surgery. We need to pull out those teeth. So he was costing me for extractions of baby teeth. But it was ridiculous and I felt like I distrust that dentist’s place. I was just like, he was I guess a crook, you know what I mean? Like I felt right away that the place just wants money, money, money, money, you know what I mean? Personally, that's how I felt - Mixed group participant (urban community)

Interactions that did not feel transparent to parents were said to compromise trust in dentists. They also created a fear of dentists, likely impacting the parent’s decision to take their child for dental visits.

## Discussion

We have learned that ECOH in FN and Metis communities is impacted by a mutually reinforcing mix of personal and social determinates of health brought on by long standing socio-economic exclusions and political inequities [32]. Prevalence and persistence of ECC rates and experiences, is consistent with other indigenous populations within Canada and elsewhere [6, 8, 33, 34].

Attempts to reduce ECC must therefore understand and address root causes as well as parental and familial agency. One of the dynamics although not expressly discussed by participants but glaringly evident, is one parenting roles and health decisions making. More females attended the focus groups. This was not intentional as everyone interested had been invited, but the poor male attendance is likely indicative of usual caregiving roles of females being responsible for children under 72 months. However, the actions or inactions of males can and do influence health behaviors in the home. ECOH promotion is needed, targeting males who may or may not be caregivers, but who could influence children or female health decision making in the household. The males in this study were knowledgeable and insightful, their viewpoints could be further explored. Other challenges and problems that must be tackled to promote ECOH, include firstly, the well-known fact that restricted access to dental care is associated with poor ECOH [11, 35]. While the concept of access has no universally accepted definition, the FN and Metis participants in this study revealed important insight into factors that currently limit access for their children and act as barriers from attaining and maintaining optimal oral health including absent social determinants of health, limited transportation, lack of adequate oral health information available and dentists’ behaviors. It appears that a three pronged approach may be necessary to address current access and equity disparities – policies, education and action. All three are suited to work together, with policy and education driving direct action to change current ECOH realities of Indigenous peoples and communities.

Inadequate access to oral hygiene instruction, oral health supporting nutrition, and information on how to access oral healthcare could result in continued inequities and disparities [36]. It has been suggested that involving non-dental professionals and Indigenous dental care workers could be effective in delivering quick access and culturally informed care [34, 37]. Culturally grounded models have been used and recommended as effective engagement and chronic disease prevention tools among American Indians [38]. Alaska Natives [39], and Australian Indigenous peoples [40].

Policies and actions could be geared toward better access to dental clinics, affordable transportation to access dental services, access to appropriate, and accurate oral health information to guide oral hygiene practices at home. Such policies and actions could also work to combat poverty that creates food insecurity and poor nutrition compromising the overall health of FN and Metis peoples. Campaigns encouraging families and other caregivers to reduce children’s intake of sugary foods and beverages could also be useful in promoting ECOH. We also learned from participants in the study, that dentists’ behaviors and seeming to suggest a lack of transparency, can harm parents and their children’s trust. Thus dentists and other dental professionals must strive to maintain transparency and build trust with parents and their children, which may encourage long term compliance of families and result in better ECOH [14].

Some participants have said, in light of rural living, that it could be easier for a dental provider to go to the community so families are not scrambling to look for a dentist and/or missing appointments due to logistical and transportation difficulties. Commuting to care and not having transportation to get there is a challenge faced by FN and Metis parents in both rural and urban communities. Limited access to transportation is known to affect access to care and impact health of people living with chronic conditions [41] and more so for remote populations [42]. Not only do some people living in rural and remote locations not have publicly funded or private transportation, but the transportation that is available might be atrociously expensive. This creates additional strain on scarce resources and causes parents to refrain from seeking dental care. Although some FN participants in this study said that they have some transportation coverage through the NIHB program, reported complications with Treaty status identification is also said to often exclude parents or their children from utilizing covered transportation services for medical appointments.

Registration with the NIHB dental program is one particularly problematic barrier for registered FN people to get their infants dental care. Currently, some young children are unable to access first dental visits by the first birthday in keeping with professional dental recommendations, because they do not have a Treaty number to confirm their FN status. The PI of this study [RJS] sees this every few months, where parents who have not been able to get their child registered, are often delayed in getting their infant in for the checkup by age 12 months.

Some participants believe that the Department of Indigenous Services Canada policies is limit access to dental care by paring down the procedures that can be covered for FN patients. Federal and provincial health policies, while well intentioned, can and do limit access to care for Indigenous populations [43–46].

Contextualized and tailored care may be the answer to policy and systemically induced inequities in healthcare systems [15, 47]. For example, Treaty status, which is a ticket to NIHB for FN, is determined by the Federal Indian Act [18]. FN people who are without status for various reasons are excluded from accessing available resources. An infant not being registered with NIHB right away, and lacking a number, can be a barrier to getting a dental appointment as the dental office cannot bill NIHB for the early preventive dental visits by 12 months of age. Delays in getting paperwork completed and processed also means that many never get seen by their first birthday, as is recommended by dental providers. Perhaps community determined Treaty status may make it easier for more community members to quickly access health benefits offered through the NIHB, including transportation. Considering the high prevalence of ECC in FN and Metis populations, this would be an important step in promoting equity in ECC prevention and access to dental care. While NIHB has observably enhanced its programs over the years to remove the need for predetermination for many procedures, many registered FN peoples may not be aware of the enhancements or that it may be easier to access care. Perhaps, NIHB also needs to undertake an education campaign with its beneficiaries so that parents and practitioners alike can truly understand their dental benefits.

Stigmatization and discrimination of beneficiaries of public funding is also unfortunately a common occurrence as some people face discrimination when they have social insurance and as when they do not [48, 49]. Some face difficulties because of presumptions made of them drawing on public funds undeservingly. On the flip side, others may not receive the best service if they are not seen to have adequate insurance. As reported by participants in this study, some healthcare providers appear less interested in the patients’ wellbeing when they find that the patient has little or no money. Education for dental professionals to promote better understanding of micro aggressions, lack of transparency and outright racism directed at Indigenous populations which erodes trust and impacts compliance is needed. This could be useful in reducing predatory behaviours as reported to be the case in some dental offices. The result could be the promotion of proactively respectful and accommodating behaviors.

Facilitated pathways to supporting access to dental care could help in the form of direct information sharing between the NIHB and dental offices so the offices are frequently made aware of any changes to the program. Better information sharing could also mean that dental offices would be more flexible in accommodating patients and making changes to appointments without penalties when families are not able to make scheduled days and/or times due to administrative challenges with NIHB. Better information sharing may also help prevent ECC by clarifying what resources are available to parents and caregivers to enhance access to dental care in general. The persistent lack of information, lack of clarity overall and sheer difficulty of navigating systems, prevents utilization. An example of how accurate information could help prevent ECC, is that some parents in this study seemed to think that costlier oral hygiene products would do a better job of cleaning their child’s teeth. Not only is this not the case but it also shows how a paucity of evidence-based, professional driven information flow to parents and caregivers can expose children to ECC when parents assume they are not able to do anything about their child’s oral health. Parents may be waiting to purchase expensive oral hygiene products when more affordable products are just as effective.

Sometimes, families with NIHB or private insurance coverage pay some fees upfront before receiving care at dental offices. Some families become discouraged by this realization and stop attending dental clinics. They need to know the expectations for out-of-pocket costs so they can plan appropriately. Once again, facilitated pathways [50] coordinated by community-based primary healthcare workers may help FN navigate available resources and help Metis patients access affordable options planned and executed with the communities.

Participants in this study mentioned that other adults make poor food and beverage choices for their children. Mass awareness of practices, products and nutritional impacts on ECOH could change the attitudes and adult oral health related behaviors around children and possibly reduce risky behaviors such as the tendency to offer sugary substances when brushing afterwards cannot be implemented (Blinded Reference). Mass awareness must also be supported by practical opportunities to make actual changes. For example, a recent study highlighted the association between quality nutrition and reducing early childhood caries [51] and others the role vitamin D could play in supporting ECOH [52, 53]. However, parents living in poverty may bypass such information if they are not able to afford healthy food options. A real opportunity is presented when such awareness is followed by a listing of essential vitamins supporting ECOH that could be easily obtained from consuming traditional foods available on Indigenous lands and territories [53]. Such information could be amply circulated in community-based settings and also to policy makers, social planners and local leadership who could provide support maternal and early childhood access to such nutrition.

While raising awareness among the communities of the availability of opportunities, such as the free dental visit for children up to 1 year in Manitoba is required, policies will also need to address the lack of capacity to meet the increased demand for visits that will be generated, and which may result in general dissatisfaction of patients to lengthy wait-times. Also, having to wait for more than 6 months for an appointment, as currently happens, is unacceptable, especially if a child already has early stages of caries which may progress to a more severe stage in that length of time. Policies that will create or facilitate care pathways may enable a faster establishment of a dental home for priority populations at higher risk of ECC.

One way to help keep parents and caregivers aware of ECOH and prevent ECC could be working in collaboration with non-dental healthcare providers [54, 55]. Families tend to interface with primary care providers more often than with dental providers, therefore, involving primary healthcare practitioners in caries assessments could increase and fast track access to dental professionals and dental care overall. Moreover, integration of oral care into primary care practice is gaining increasing appreciation and could be successfully implemented in community-based contexts with some management of information and roles [56]. Integration in this way, is important for holistic health and is often advocated for by Indigenous peoples [57, 58] Thus in partnership with Public Health Agency of Canada, our team developed the Canadian Caries Risk Assessment Tool for use by non-dental healthcare professionals or dental professionals in non-dental settings (https://umanitoba.ca/CRA_Tool_ENG_Version.pdf). This tool can be used to identify children at high risk for caries, provide anticipatory guidance for parents, and establish a pathway of referral for those who may need additional dental care. Perhaps the use of technology to perform dental screenings remotely using the tool could be considered, especially now given a new propensity for conducting healthcare via use of technology.

### Study limitations

Our research team includes Indigenous community members, Indigenous community leadership, (including FNHSSM and MMF), health professionals, local, provincial and national decision-makers and academics. This team structure promoted the sharing of recommendations with stakeholders as data became available. Results have also been shared with FN and Metis organizations and communities in formats that can promote further discussions and implementation plans. We however note that a stronger distinction could be made of the experiences and factors affecting ECOH among urban versus rural FN and Metis peoples. Our analysis did not explicitly consider these differences. Those differences will be considered in future analyses. The results may also not necessarily be generalizable to every FN and Metis community in Canada, thus the recommended strategies can be expanded by conducting additional studies with more communities and with increased sample size.

## Conclusion

Closing existing ECOH gaps for FN and Metis peoples and communities requires equity-oriented healthcare that addresses specific problems and challenges faced by this population. Solutions to family, community and systemic level issues seem commonsensical and easy to accomplish but have persisted. We have learned that the solutions may lie close to or within the problems themselves. In sum, policy makers, funders and dental professionals (policy) can facilitate access to dental care for Indigenous peoples by providing adequate and appropriately delivered information (education) to impart evidence-based ECOH practices for parents, caregivers and dental practitioners alike (action). Widespread family-inclusive ECOH education could mediate risky parental and caregiver behaviors. Increasing access to dental care could be accomplished by resolving bureaucratic processes in determining FN Treaty status and insurance difficulties faced by FN and Metis alike. Better information on what resources are available to families along with easier systems navigation pathways and access to funded transportation for rural and remote dwelling patients may encourage more families to attend dental appointments. Affordable and easily acquired food options should also closely follow on the heels of nutrition education for families. Finally, the education of dental professionals on the unique barriers to ECOH faced by FN and Metis peoples and communities and their roles in entrenching the barriers knowingly or unknowingly cannot be overstated. Ultimately, access and equity must remain a central goal in preventing ECC and promoting ECOH among Indigenous peoples.

## Data Availability

Not applicable.
